# Low neuronal metabolism during isoflurane-induced burst suppression is related to synaptic inhibition while neurovascular coupling and mitochondrial function remain intact

**DOI:** 10.1177/0271678X211010353

**Published:** 2021-04-25

**Authors:** Nikolaus Berndt, Richard Kovács, Karl Schoknecht, Jörg Rösner, Clemens Reiffurth, Mathilde Maechler, Hermann-Georg Holzhütter, Jens P Dreier, Claudia Spies, Agustin Liotta

**Affiliations:** 1 Institute for Imaging Science and Computational Modelling in Cardiovascular Medicine Charité – Universitätsmedizin Berlin, corporate member of Freie Universität Berlin, Humboldt-Universität zu Berlin and Berlin Institute of Health, Berlin, Germany; 2 Institute for Neurophysiology, Charité – Universitätsmedizin Berlin, corporate member of Freie Universität Berlin, Humboldt-Universität zu Berlin and Berlin Institute of Health, Berlin, Germany; 3 Carl-Ludwig-Institute for Physiology, University Leipzig, Leipzig, Germany; 4 Neuroscience Research Center, Charité – Universitätsmedizin Berlin, corporate member of Freie Universität Berlin, Humboldt-Universität zu Berlin and Berlin Institute of Health, Berlin, Germany; 5 Center for Stroke Research Berlin, Charité – Universitätsmedizin Berlin, corporate member of Freie Universität Berlin, Humboldt-Universität zu Berlin, and Berlin Institute of Health, Berlin, Germany; 6 Department of Experimental Neurology, Charité – Universitätsmedizin Berlin, corporate member of Freie Universität Berlin, Humboldt-Universität zu Berlin, and Berlin Institute of Health, Berlin, Germany; 7 Institute of Biochemistry, Charité – Universitätsmedizin Berlin, corporate member of Freie Universität Berlin, Humboldt-Universität zu Berlin and Berlin Institute of Health, Berlin, Germany; 8 Department of Neurology, Charité – Universitätsmedizin Berlin, corporate member of Freie Universität Berlin, Humboldt-Universität zu Berlin, and Berlin Institute of Health, Berlin, Germany; 9 Bernstein center for Computational Neuroscience, Charité - Universitätsmedizin, Humboldt-Universität zu Berlin and Technische Universität Berlin, Berlin, Germany; 10 Einstein Center for Neuroscience, Charité - Universitätsmedizin Berlin, the Freie Universität Berlin, the Humboldt-Universität zu Berlin and the Technische Universität Berlin, Berlin, Germany; 11 Department of Anesthesiology and Intensive Care, Charité – Universitätsmedizin Berlin, corporate member of Freie Universität Berlin, Humboldt-Universität zu Berlin and Berlin Institute of Health, Berlin, Germany; 12 Berlin Institute of Health, Charité – Universitätsmedizin Berlin, corporate member of Freie Universität Berlin, Humboldt-Universität zu Berlin and Berlin Institute of Health, Berlin, Germany

**Keywords:** Anaesthesia, burst suppression, cerebral blood flow, isoflurane, mitochondria

## Abstract

Deep anaesthesia may impair neuronal, vascular and mitochondrial function facilitating neurological complications, such as delirium and stroke. On the other hand, deep anaesthesia is performed for neuroprotection in critical brain diseases such as status epilepticus or traumatic brain injury. Since the commonly used anaesthetic propofol causes mitochondrial dysfunction, we investigated the impact of the alternative anaesthetic isoflurane on neuro-metabolism. In deeply anaesthetised Wistar rats (burst suppression pattern), we measured increased cortical tissue oxygen pressure (p_ti_O_2_), a ∼35% drop in regional cerebral blood flow (rCBF) and burst-associated neurovascular responses. *In vitro*, 3% isoflurane blocked synaptic transmission and impaired network oscillations, thereby decreasing the cerebral metabolic rate of oxygen (CMRO_2_). Concerning mitochondrial function, isoflurane induced a reductive shift in flavin adenine dinucleotide (FAD) and decreased stimulus-induced FAD transients as Ca^2+^ influx was reduced by ∼50%. Computer simulations based on experimental results predicted no direct effects of isoflurane on mitochondrial complexes or ATP-synthesis. We found that isoflurane-induced burst suppression is related to decreased ATP consumption due to inhibition of synaptic activity while neurovascular coupling and mitochondrial function remain intact. The neurometabolic profile of isoflurane thus appears to be superior to that of propofol which has been shown to impair the mitochondrial respiratory chain.

## Introduction

In neurons, about 50% of the produced adenosine triphosphate (ATP) is required for maintaining the transmembrane ion gradients and for neurotransmission.^1^ Anaesthetics are known to reduce brain metabolism and performing deep anaesthesia is a therapeutic strategy to achieve neuroprotection in the course of severe brain disease such as a status epilepticus or traumatic brain injury (TBI).^2^,^3^ On the other hand, it has been claimed that deep anaesthesia is neurotoxic and could contribute to postoperative neurological complications.^4–7^ In particular, anaesthetic-induced changes in neurovascular coupling and mitochondrial function could facilitate postoperative brain dysfunction after surgery.^4–6,8,9^ Anaesthesia depth can easily be assessed using electroencephalography (EEG). Cortical alpha/delta activity corresponds to the phase 2, the typical state of general anaesthesia, while the burst suppression pattern (phase 3) corresponds to deep anaesthesia. The electrophysiological signature of burst suppression is characterized by cyclic isoelectric periods interrupted by paroxysmal bursting activity in the alpha/beta range.^10^ Importantly, burst suppression also characterizes severe metabolic brain dysfunction.^11^,^12^ In this line, it has been proposed that both brain disease and pharmacologically induced burst suppression might occur due to periodic ATP-decreases and consequent activation of K^+^ dependent ATP-channels.^7^ As anaesthetics might differently influence neuro-metabolism during burst suppression, the characterization of their specific effects might help clinicians to choose the appropriate anaesthetic. This might apply in particular when anaesthesia is required in patients with cerebral injuries or other brain diseases.

The halogenated gas isoflurane is used to perform anaesthesia during surgery, for sedation in the course of critical care and to control seizures during super-refractory status epilepticus.^2^ However as gas anaesthetics are thought to increase intracranial pressure, the use of isoflurane in neurocritical care and neurosurgery has become rare.^13^ Isoflurane has a plethora of effects on neurons: change of the fluidity of the lipid bilayer, activation of GABA_A_-receptors, inhibition of NMDA-receptors, opening of two-pore domain K^+^ channels and blocking of presynaptic vesicle exocytosis via Ca^2+^ channels.^14–17^ As synaptic processes account for a significant portion of the ATP demand, the inhibition of neurotransmission during deep anaesthesia might increase intracellular ATP concentration with secondary reduction of ATP production.^7,14^ In addition, isoflurane could alter substrate availability by effects on neurovascular coupling and vascular autoregulation.^8^ Moreover, it has been hypothesized that isoflurane directly affects ATP synthesis in mitochondria.^15–17^ In this line, opening of the mitochondrial transition pore and a direct inhibition of the mitochondrial complex I has been described.^17,18^ Particularly, the inhibition of the complex I at presynaptic terminals has been proposed to play a role in isoflurane associated inhibition of neurotransmission.^17^,^18^ In contrast, lower levels of O_2_ and intracellular ATP decay with concomitant rise in lactate has been observed under propofol administration, while isoflurane reduced energy demand, decreased lactate and increased ATP availability without direct alterations of the respiratory chain in the brain of anaesthetised swine.^19^

We have shown previously, that propofol generates changes in mitochondrial redox state compatible with a direct inhibition of the complex II.^20^ In the present study, we sought to elucidate changes in neuro-metabolism during deep isoflurane anaesthesia by combining *in vivo* and *in vitro* measurements with a computational modelling approach. *In vivo,* we measured cortical p_ti_O_2_ and rCBF during phase 2 and burst suppression anaesthesia. *In vitro*, we combined local field potential (f.p.) and ion-selective- as well as O_2_ electrode recordings with intracellular Ca^2+^ and flavin adenine dinucleotide (FAD) imaging. We used FAD fluorescence as a direct marker for the mitochondrial redox state.^23,24^ Based on the experimental data, we then performed computer simulations with the aim to discriminate between direct and indirect effects of isoflurane on mitochondrial function.^29–31^

## Materials and methods

This study complies with the ARRIVE 2.0 guidelines, the Helsinki declaration and the Charité animal welfare guidelines. The experimental protocols were approved by the State Office of Health and Social Affairs of Berlin (G0264/14 & T0096/02). Prior to *in vivo* experiments or tissue extraction for the *in vitro* experiments, the animals had at least 7 days for acclimation in our animal shed. Accommodation was in groups of two with food ad libitum and a 12-h light on light off cycle.

### In vivo recordings of cortical p_ti_O_2_ and rCBF under deep isoflurane anaesthesia

14 male Wistar rats (weight:∼250 g, age: 8 ± 1 weeks) were place in closed chamber and anaesthesia was induced with isoflurane and nitrous oxide (N_2_O) (1.5% and 70% respectively). 1 animal was excluded from the study due to excessive bleeding in the recording area. Following induction, anaesthesia was continued with 1-2% isoflurane (without N_2_O) and 50% fraction of inspiratory O_2_ through a nares-mask. Pulse oximetry was monitored (MouseOxplus®, Starr life Sciences, Oakmont, USA) during the surgical procedure (total duration of preparation procedure was ∼90 ± 20min). Body temperature was maintained at 37.0 ± 0.5 °C (Harvard Apparatus, Holliston, USA). Animals were mechanically ventilated after tracheotomy (Harvard Small Animal Ventilator 683, Holliston, USA). End-tidal CO_2_ (ETCO2) was monitored and maintained at ∼35 mmHg and FiO_2_ was maintained at 30% during recordings. Blood pressure was continuously (femoral artery). A mini craniotomy in the frontal region 2 mm from the sagittal suture and 2 mm from the coronal suture was performed after fixation in a stereotaxic system. After dura incision, a microelectrode for electrocorticography (ECoG) and a Clark-type O_2_ electrode (tip: 10 µm; Unisense, Aarhus, Denmark) were inserted in the frontal cortex at a depth of ∼100µm. For rCBF measurements and assessment of neurovascular coupling, an ECoG electrode and a double-wired stimulation-electrode were inserted into the cortex, while a laser Doppler probe (Optronix, OxyFlo™2000) was placed 0.2–0.3 mm above the cortical surface. Direct cortical stimulation (duration: 2 s, frequency: 20 Hz, intensity: 5 V) was performed at a distance of 3.5 mm from the laser Doppler probe. A control period of approximately 10–20 min was recorded in phase 2. Afterwards, burst suppression was established and maintained for approximately 60 min (total recording time ∼100 min). After recordings, the animals were sacrificed in deep anaesthesia, brain tissue was removed and flash frozen for future analysis.

### Slice preparation and maintenance

For *in vitro* experiments, hippocampal slices were prepared from 27 male Wistar rats (weight: 250 g, age: 8 ± 1 weeks) as previously described.^20^ Artificial cerebrospinal fluid contained (in mM): 129 NaCl, 21 NaHCO_3_, 10 glucose, 3 KCl, 1.25 NaH_2_PO_4_, 1.6 CaCl_2_, and 1.8 MgCl_2_. Osmolarity was 295–305mosmol/L and pH was 7.35-7.45. As circuitry and cell distribution is well known, the area CA3 of the hippocampal formation was chosen to perform electrophysiological and imaging experiments concerning changes in metabolism and underlying alteration in synaptic transmission/network oscillations.^21^ Furthermore, the generation of network oscillations in the hippocampal formation is an established method to study active cell assemblies and metabolic changes.^20^,^22^,^23^

### Electrophysiology, p_ti_O_2_ recordings and fluorescence imaging in vitro

Glass microelectrodes filled with saline (154 mM NaCl) were used for f.p. recordings placed in the stratum pyramidale in area CA3. Electrical stimulation (bipolar electrode) was applied in stratum radiatum of area CA1. Stimulation consisted in paired pulses (100 µs duration, single pulse interval 50 ms, paired pulse interval 60 s). During FAD, p_ti_O_2_, extracellular Ca^2+^ ([Ca^2+^]_o_) and extracellular K^+^ concentration ([K^+^]_o_) recordings, neuronal activation was induced by 2 s long 20 Hz tetani (single pulse duration 100µs, interval 50 ms, 40 pulses) every 10 min as previously described.^21^ Simultaneous f.p, p_ti_O_2_. and [K^+^]_o_ or [Ca^2+^]_o_ measurements were performed using double-barrelled ion-sensitive microelectrodes constructed and calibrated as reported.^21^ The O_2_ electrode was moved vertically through the slice in 20 μm steps until reaching the minimum of p_ti_O_2_. FAD autofluorescence imaging was performed in area CA3 with a 20x Objective (numerical aperture 0.5) using a custom-built setup equipped with a light emitting diode (LED, 460 nm wavelength, Lumen, Prior scientific, Seefelder, Germany) and a photomultiplier tube (PMT, Seefelder Messtechnik). Simultaneous [Ca^+2^]_o_ measurements and imaging of intracellular Ca^2+^ fluorescence was performed with the same setup configuration after staining with the AM-ester form of Oregon Green 488 BAPTA-1 (OGB-1, Bioscience, USA).

### Isoflurane application and induction of gamma oscillations

Isoflurane was applied in the interface system or dissolved in aCSF (submerged condition) together with carbogen using a calibrated isoflurane vaporizer (Dräger, Germany) at a gas flow of 1 l/min. Concentration of isoflurane was controlled using a Vamos® mobile isoflurane monitor (Dräger, Germany). The recording chamber temperature was ∼36°C for both the interface and submerged conditions. Thus, taking in account a water/gas partition coefficient for 37 °C of 0.54,^24^ the application of 1% and 3% correspond to 0.24 and 0.72 mM isoflurane in the aCSF.^25^ Gamma oscillations were induced by application of 10 µM acetylcholine chloride and 2 µM physostigmine salicylate (Sigma-Aldrich, Steinheim, Germany).

### Data acquisition and data analysis

Analog signals were digitalized with a Power CED1401 and Spike2 software (Cambridge Electronic Design, Cambridge, UK). Data analysis and statistics were performed using Spike2, Excel (Microsoft, Seattle, USA) and Origin (Version 6, Microcal Software, Northampton, USA). The median values and corresponding 25^th^ and 75^th^ percentile in brackets are described in results. Data is shown in dot plots with median or, in the case of time lines, in mean ± standard deviation (SD). Fluorescence of FAD or OGB-1 are shown as Δf/f_0_ where f_0_ is the average of 15 s baseline prior to stimulation. Fluorescence decay was analysed by normalization of 5minutes prior to stimulation in the control, under treatment and after wash out of isoflurane (f_0_ was the measured first point). For statistical comparison, the percent decay after 5minutes was compared. Power spectra of gamma oscillations were calculated with Spike2 (5minutes per condition, FFT, Hanning window, size 4096). For statistical inference, we first tested for normal distribution using the Kolmorogov-Smirnov test followed by paired Student’s t-test. When multiple comparison was performed, p values were adjusted using the Bonferroni-correction. Changes were stipulated to be significant for p-values <0.05.

### Calculation of cerebral metabolic rate of O_2_

Calculation of cerebral metabolic rate of O_2_ (CMRO_2_) was calculated from p_ti_O_2_ depth profiles as previously described.^20^ In short, we applied a reaction-diffusion model for O_2_ consisting of diffusive O_2_-transport and O_2_-consumption within the slice. Slices were divided into layers with equal thickness of 1 μm. Diffusive distribution of O_2_ between the layers is described by Fick’s Law with a diffusion constant of 1.6 × 103 μm^2^/s and O_2_ consumption rate within each layer is given by Michaelis-Menten kinetics with a Km-value of 3 mmHg.^24^ The CMRO_2_ was assumed to be homogeneous throughout the slice and is treated as an adjustable parameter to match the experimental data. For the boundary conditions, the p_ti_O_2_ concentration at the slice surface was fixed to the supply value, while at the p_ti_O_2_ minimum the diffusive transport of O_2_ was put to zero.

### Calculations of FAD transients and ATP consumption rates

As alterations in FAD fluorescence originate from the pyruvate dehydrogenase (PDHC), the α-ketoglutarate dehydrogenase (KGDHC), the glycerol-3-phosphate dehydrogenase (G3PDH) and the succinate dehydrogenase (SUCCDH) complexes, fluorometric measurements of FAD permits to study mitochondrial redox state.^24–28^ Based on calculated CMRO_2_, we used the metabolic model of neuronal energy metabolism to simulate stimulus-induced FAD transients and ATP consumption rates as established and described by Berndt et al. 2015.^25^ Differences in basal CMRO_2_ during isoflurane administration imply differences in basal ATP consumption rates, as increased ATP consumption lowers ATP levels, activates glycolysis, citric acid cycle and respiratory chain activity and concomitantly increases CMRO_2._ Isoflurane-induced changes in basal ATP consumption rates were simulated by adaptation of the resting ATP demand to match the observed resting CMRO_2_. In experiments with electrical stimulation, we simulated the time-dependent metabolic response to a brief stimulus-induced increased ATP demand and corresponding cytosolic calcium transient in addition to the isoflurane dependent changes in metabolic resting state. The time course of the energetic load, i.e., the increase in the ATP demand associated with the activating stimulus was described by a rectangular activation function describing a short period of high metabolic demand (corresponding to the duration of stimulation), while the associated cytosolic Ca^2+^ transient was modelled as steep Ca^2+^ increase (corresponding to sudden stimulus-induced Ca^2+^ influx into the cell) followed by a slowly decaying component (corresponding to the slower pumping of Ca^2+^ from the cytosol out of the cell). The magnitude of the stimulus was set by taking in account the calculated CMRO_2_ in control and after 3% isoflurane. Cytosolic Ca^2+^ is rapidly taken up into mitochondria by Ca^2+^ uniporter. The Ca^2+^ taken up by the mitochondria is first sequestered by Ca^2+^-binding proteins and then released into the mitochondrial matrix where it activates the mitochondrial dehydrogenases PDHC, isocitrate dehydrogenase, and KGDHC. At the same time, increased ATP demand decreases cytosolic ATP levels thereby activating glycolysis, cellular shuttle systems and increasing mitochondrial activity. The corresponding changes in the reduction state of protein bound FAD moieties are then compared to the observed FAD fluorescence changes obtained by the fluorometric measurements for validation. For all simulations we used MATLAB Release2012a (The MathWorks, Inc., Natick, MA, USA) with the optimization toolbox.

## Results

### Burst suppression anaesthesia is associated with increased p_ti_O_2_, lower rCBF and intact neurovascular responses

**Figure 1.**
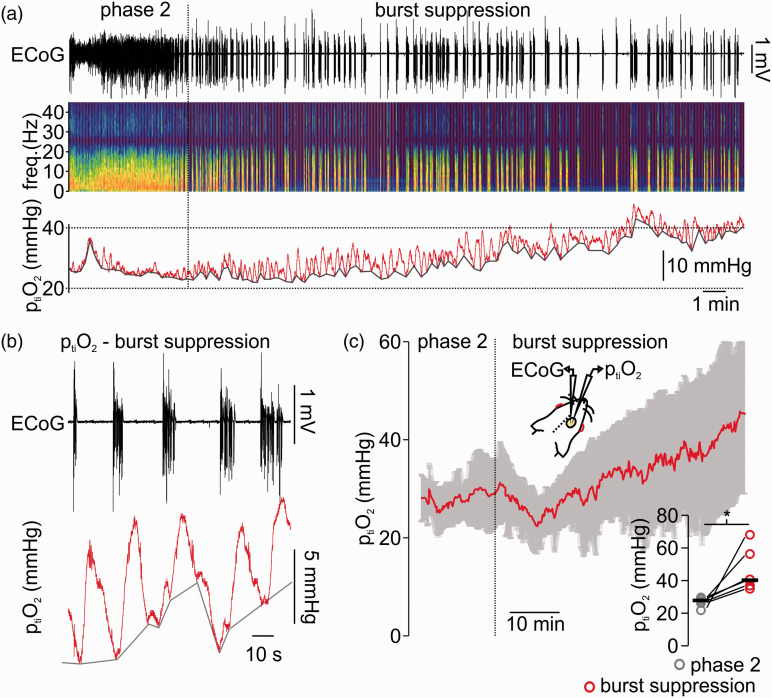
Burst suppression anaesthesia with isoflurane is related to tissue partial pressure of oxygen (p_ti_O_2_) increase and burst associated O_2_-fluctuations. (a) Sample recording of simultaneous electrocorticography (ECoG) and tissue oxygen (p_ti_O_2_) during light anaesthesia (i.e. phase 2 on the left) and during induction of burst suppression (right part of the trace). As shown in the ECoG trace and corresponding spectrogram, phase 2 anaesthesia is characterized by ongoing activity at delta/alpha frequency ranges while burst suppression is typically a succession of isoelectric moments interrupted by bursts. The p_ti_O_2_ baseline increased and burst-coupled p_ti_O_2_ fluctuations occurred (trace on top, ECoG-trace in black, in the middle spectrogram and at the bottom p_ti_O_2_ trace in red). (b) Detail of the recording shown in (a) displaying the typical pattern of burst suppression (ECoG trace on top, black) and burst-associated p_ti_O_2_-changes (trace in bottom, red). (c) Time-line plot of averaged p_ti_O_2_ when isoflurane anaesthesia was transformed from phase 2 to burst suppression (n = 6, red line average, in grey SD). Right corner, single values (circles, grey: phase 2 and red: burst suppression, black lines: median) of averaged p_ti_O_2_ during phase 2 and after 40 minutes of burst suppression (10 minutes average, n = 6, paired t-test). *=p < 0.05.

To characterize changes in p_ti_O_2_ under deep isoflurane-anaesthesia, we first performed simultaneous recordings of ECoG and p_ti_O_2_ in anaesthetised rats. We analysed both, changes in p_ti_O_2_ baseline and dynamic fluctuations during burst suppression (see methods). During the period of phase 2 anaesthesia (characterized by delta/alpha activity in the ECoG), the averaged p_ti_O_2_ was 27.8 (26.1,28.0)mmHg and increased to 40.2 (36.8,56.2)mmHg after 40 minutes of burst suppression (p = 0.02, n = 6, Figure 1(a) and (b)). This increase in p_ti_O_2_ baseline was accompanied by burst-associated local p_ti_O_2_ increases of ∼5mmHg (Figure 1(b)). These burst-coupled p_ti_O_2_ increases started simultaneously with the burst and reached a peak during the inter-burst period with a latency of ∼3 s to the burst onset. Thus, the changes in p_ti_O_2_ during deep anaesthesia had two components, 1st: increase in p_ti_O_2_ baseline and 2nd: local, burst-associated p_ti_O_2_ fluctuations. Since p_ti_O_2_ in the brain depends on the O_2_ transport by erythrocytes, vascular tone and O_2_ consumption within the brain tissue, we asked whether the observed alterations in p_ti_O_2_ during isoflurane-induced burst suppression might be due to changes in rCBF.

Since O_2_ transport is performed by erythrocytes and depends on changes in vascular tone, we asked whether the observed alterations in p_ti_O_2_ during isoflurane-induced burst suppression might be due to changes in rCBF.

**Figure 2.**
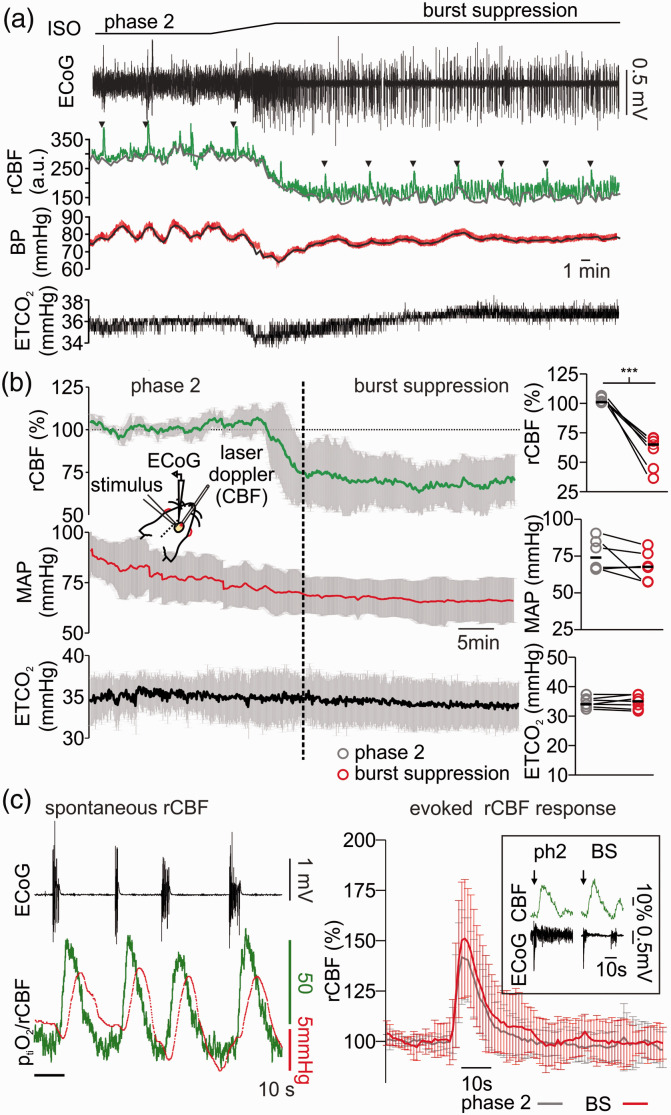
Changes in regional cerebral blood flow (rCBF) during light and deep anaesthesia with isoflurane. (a) Exemplary experiment of simultaneous electrocorticography (ECoG) and laser Doppler flowmetry displaying changes in rCBF in dependence of anaesthesia depth (ECoG trace on top, black and laser Doppler flowmetry middle, green). Systemic blood pressure (BP, red trace in the middle) was invasively measured during the recordings and end-tidal carbon dioxide (ETCO_2_, black trace on bottom) was monitored and adjusted to constant concentrations of ∼35mmHg by modifications of the mechanical ventilation. During induction of burst suppression by increasing the inspiratory fraction of isoflurane (from ∼1.0% to ∼3.0%), the baseline rCFB decreased and reach a steady state that was ∼35% below the rCBF during phase 2 anaesthesia. (b) Time line plot (average and SD) of 6 *in vivo* recordings concerning changes of rCBF (green line, on top), middle arterial pressure (MAP, red line in the middle) and ETCO_2_ (ETCO_2_) measured during phase 2 and burst suppression anaesthesia. Right, bar plots of single experiments values (10 minutes averages for each condition) of rCFB, MAP and ETCO_2_ during phase 2 and burst suppression (grey and red respectively). (c) Left: detail of a simultaneous rCBF and p_ti_O_2_ recording (performed in one animal) during burst suppression. Bursts (ECoG, black trace on top) were followed by spontaneous increases in rCBF (green trace) and consecutive increases in cortical p_ti_O_2_ presumably due to intact neurovascular coupling during deep anaesthesia with isoflurane. Right: plot of averaged stimulus induced rCBF answers after a 2 s 20 Hz stimulus train during phase 2 (black line) and burst suppression (red line) and, framed, example traces of stimulus-induced (black arrows) rCBF changes during phase 2 and burst suppression anaesthesia (as shown in ECoG trace, black). In (b) (plots on the right), statistical data presented in averages (circles) and medians (black lines), n = 6, significance tested with paired t-test, ***=p < 0.001.

Thus, we performed laser-Doppler flowmetry (LDF) and assessed changes in baseline rCBF, during spontaneous rCBF fluctuations coupled to burst-isoelectricity cycles and during electrical stimulus trains (see methods and Figure 2). When anaesthesia was deepened from phase 2 to burst suppression, a significant decrease of ∼35% in rCBF baseline occurred (decrease from 101.0 (100.9,100.5) to 65.0 (61.5,70.6)%, p < 0.001, n = 7). During these recordings, we monitored arterial pressure and maintained end-tidal CO_2_ (ETCO2) at ∼35mmHg by correcting the ventilation if necessary (median during phase 2 was 34.0 (33.0, 35.8) mmHg and during burst suppression 35.0 (33.8,37.3)mmHg (p = 0.7, n = 7). During deep anaesthesia, mean arterial pressure (MAP) decreased from 74.0 (66.2,84.8) during phase 2 to 67.7 (57.8,76.6)mmHg during burst suppression (p = 0.1, n = 6, one animal could not be measured due to technical issues). However, MAP always remained within the physiological range that allows autoregulation of rCBF (Figure 2(a) and (b)).^28^ To assess possible changes in neurovascular coupling, both spontaneous and stimulus-induced changes in rCBF were analysed. After single burst activity, short periods of hyperaemia were observed. In one experiment, simultaneous p_ti_O_2_ and LDF measurements corroborated that the p_ti_O_2_ increases associated to burst activity observed in previous experiments (Figure 1) were the result of a spontaneous, activity-dependent increase in rCBF (Figure 2(c)). Furthermore, electrical stimulus-evoked hyperaemia during deep anaesthesia slightly increased from 140.1(135.0,165.5)% during phase 2 to 157.4 (138.1,174.5)% during phase 3(p = 0.03, n = 7, Figure 2(c)). To further understand specific changes in the metabolism of neurons independently of fluctuations of rCBF and p_ti_O_2_, we studied the effects of isoflurane in brain slices under constant supply of O_2_ and glucose.

### Isoflurane affects synaptic transmission and diminishes CMRO_2_ in brain slices

**Figure 3.**
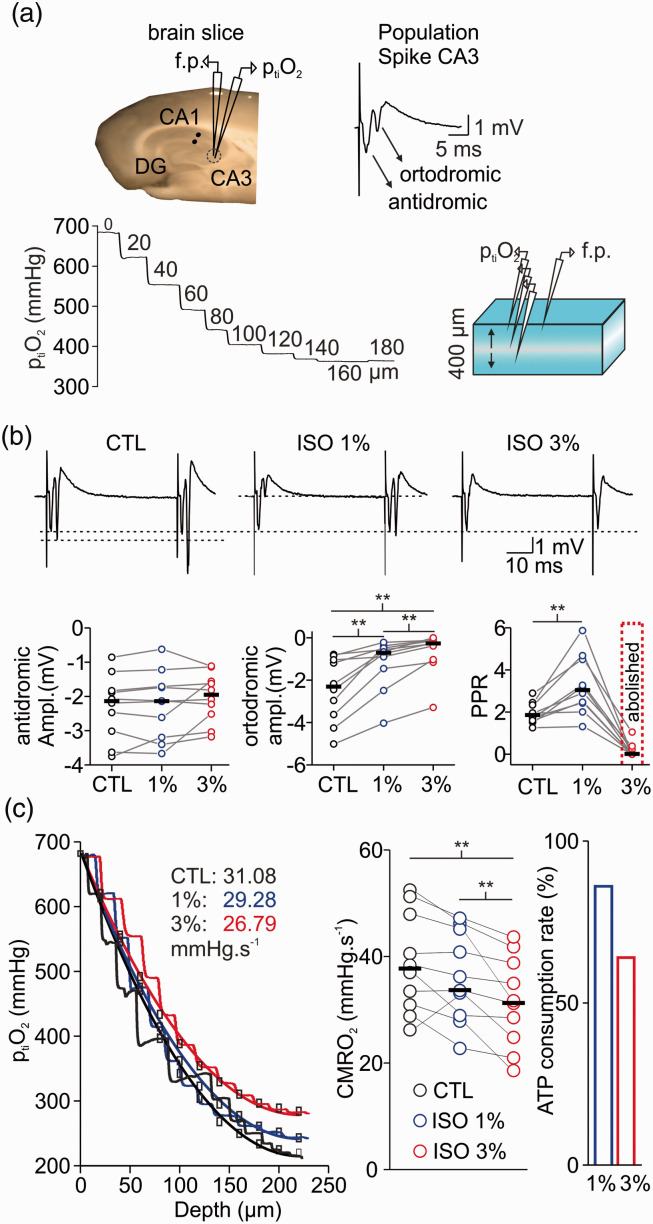
Isoflurane simultaneously affects synaptic transmission and cellular respiration *in vitro*. (a) Left: schematic drawing showing the position of the field potential electrode (f.p.), stimulation electrode (black dots) and p_ti_O_2_-Clark-style electrode in the hippocampal slice. Right: example trace of population spike (PS) in area CA3 with antidromic and orthodromic components. Bottom: representation of brain slice (right) during measurements of vertical p_ti_O_2_-gradients (left) to assess CMRO_2_ under constant O_2_ and glucose supply. (b) Top: examples traces of paired-pulse facilitation during treatment with isoflurane (ISO, top). Bottom: The orthodromic PS (left) was decreased/abolished under 1% and 3% isoflurane respectively. Interestingly, the antidromic PS remained intact (middle). Paired-pulse facilitation increased under 1% but was abolished under 3% isoflurane (right) suggesting strong inhibition of presynaptic processes. (c) Left: Overlay of p_ti_O_2_-profiles in control (CTL, black), under 1% (blue) and 3% (red) isoflurane. Treatment with 3% isoflurane (plot on the right) significantly reduced CMRO_2_ and ATP consumption by 40% as calculated based on experimental data (left). Statistical comparison with paired t-test with Bonferroni correction, **=p < 0.01, n = 10.

*In vitro,* we used 1% and 3% isoflurane to mimic the conditions of phase 2 and burst suppression, respectively. Depth profiles of p_ti_O_2_ and stimulus-induced changes in neurotransmission were simultaneously recorded under control conditions and during treatment with isoflurane in stratum pyramidale of the CA3 area (Figure 3). Calculations of changes in CMRO_2_ were performed using a reaction-diffusion model fitted to the experimentally measured p_ti_O_2_ gradients (Figure 3(a) and (c)). From 37.7 (31.0,48.0)mmHg.s^−1^ under control conditions, the calculated CMRO_2_ first decreased insignificantly to 33.7 (29.0,45.1)mmHg.s^−1^ under 1% isoflurane and then significantly to 31.2 (24.8,38.8)mmHg.s^−1^ under 3% isoflurane (1% isoflurane: p = 0.28; 3% isoflurane: 0.004; n = 10). Using a kinetic model of neuronal energy metabolism, this decrease in CMRO_2_ during isoflurane treatment corresponds to a decrease in ATP consumption by 16% and 38% at 1% and 3% isoflurane, respectively. In parallel, synaptic transmission and transmitter release probability were impaired dose dependently (Figure 3(b)). Stimulation of the Schaffer collaterals in stratum radiatum of area CA1 induces an antidromic population spike (PS) preceding an orthodromic PS in stratum pyramidale of area CA3. The antidromic PS results from direct (i.e. non- synaptic) stimulation of the CA3 pyramidal cell axons and the orthodromic PS reflects the synaptic transmission within the CA3 network via recurrent connections.^26^ In our measurements, the antidromic PS remained almost unchanged during isoflurane treatment (control: −2.13 (−3.0, −1.8)mV, 1% isoflurane: −2.14 (−3.2, −1.7)mV and 3% isoflurane: −1.95 (−2.5, −1.5)mV; 1% isoflurane: p = 0.1; 3% isoflurane: 0.07; n = 10). Furthermore, the orthodromic PS significantly decreased under 1% isoflurane and was almost abolished under 3% isoflurane (control: −2.3 (−3.5,2.5)mV, 1% isoflurane: -0.7 (−1.4, −0.5)mV and 3% isoflurane: -0.26 (−1.0, −0.1)mV; 1% isoflurane: p = 0.001; 3% isoflurane: <0.001; n = 10). To differentiate changes in pre- and postsynaptic processes, we applied paired pulse stimulation (2 pulses with an interval of 50 ms) and analysed changes in the paired pulse ratio (PPR). In the presence of 1% isoflurane, the PPR significantly increased from 1.85 (1.6,2.3) in the control to 3.0 (2.4,4.5, p = 0.001, n = 10). Conversely, 3% isoflurane almost abolished paired-pulse facilitation (p < 0.001, n = 10).

**Figure 4.**
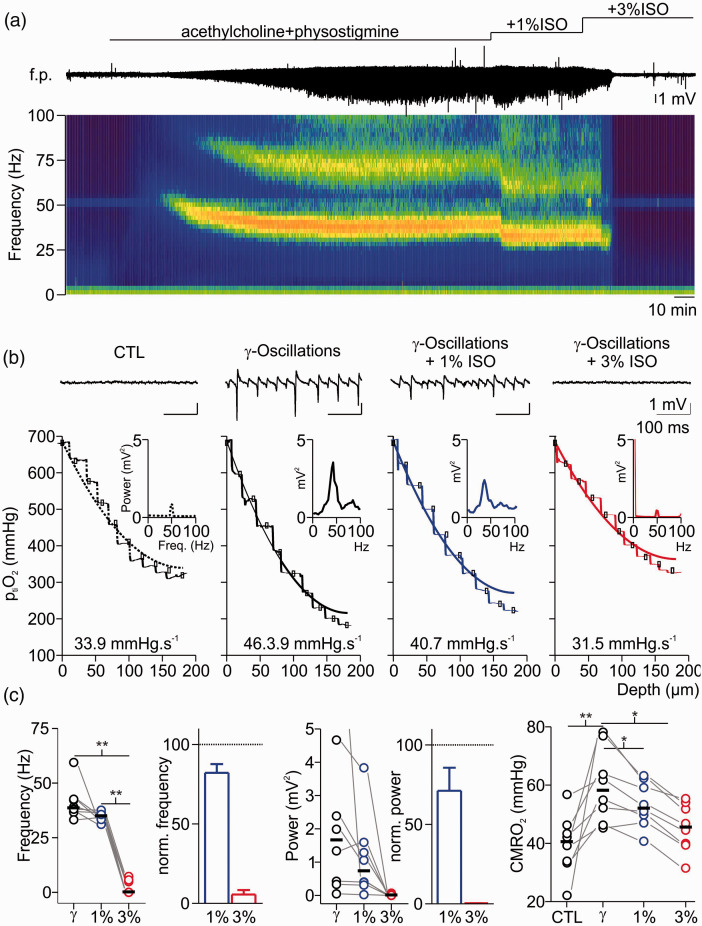
Effects of isoflurane on gamma oscillations and concomitant changes in cerebral metabolic rate of O_2_ (CMRO_2_). (a) Exemplary recording of acetylcholine (ACh) and physostigmine (Phys) induced gamma oscillations under control condition (CTL) and after treatment with 1% and 3% isoflurane (ISO). Simultaneously, interstitial partial O_2_ pressure (p_ti_O_2_, trace on top) was monitored and p_ti_O_2_-steps were performed for each condition. Application of ACh and Phys induced network activity in the gamma range as recorded in the field potential (f.p., middle trace) and confirmed by spectrogram (bottom). Application of 1% isoflurane impaired network oscillations in frequency and power, while an additional increase to 3% isoflurane completely abolished oscillatory activity. (b) Details of the same experiment showing traces before and after gamma oscillations as well as after treatment with 1-3% isoflurane (f.p. traces on top, corresponding power spectra in the middle and corresponding p_ti_O_2_-profile on bottom (dotted line: control, black line: gamma oscillations, blue line: 1% and red line 3% isoflurane respectively). (c) Plots of absolute and normalized changes in gamma frequency (left), power of peak frequency (middle) and absolute CMRO_2_ (right) under different experimental conditions. For experimental data, statistical comparison with paired t-test and Bonferroni correction, *=p < 0.05, **=p < 0.01, n = 8.

As isoflurane-induced inhibition of synaptic transmission might be the main reason for the decrease in CMRO_2_, we next addressed the question whether the effects on neuronal metabolism might be proportional to the effects on synaptic activity. To this end, we compared the CMRO_2_ during pharmacologically induced gamma oscillations under 1% and 3% isoflurane (Figure 4). These network oscillations are dependent on fast spiking GABAergic interneurons and are related to high energy demand.^20^,^23^ Application of 1% isoflurane slightly decreased gamma power and frequency (control: 1.6 (0.4,2.4)mV^2^/38.5 (36.9,42.2)Hz and 1% isoflurane: 0.73 (0.4,1.3)mV^2^/34.9 (33.7,35.8)Hz, gamma power: p = 0.6; gamma frequency: 0.2; n = 8)) and 3% isoflurane almost abolished oscillations. The computed CMRO_2_ increased from 40.7(33.9,43.3)mmHg*s^−1^ in control to 58.3 (52.2,63.7)mmHg*s^−1^ during gamma oscillations and decreased to 52.2 (49.7,59.1)mmHg*s^−1^ under 1% and to 45.7 (39.9,51.8)mmHg*s^−1^ under 3% isoflurane (p = 0.04, 0.04 and 0.002 respectively, n = 8). Thus, the treatment of slices with highly concentrated isoflurane induced severe synaptic impairment and abolishment of gamma oscillations which correlated with a decrease in oxidative metabolism.

### Changes in mitochondrial redox state are triggered by synaptic inactivation

**Figure 5.**
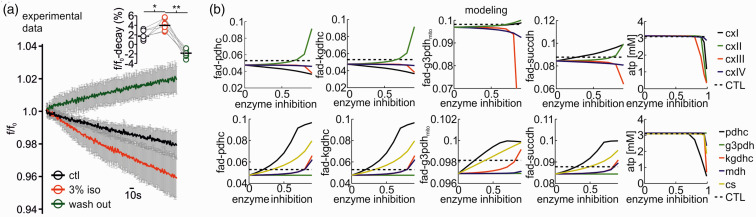
Isoflurane induced changes in redox state of FAD and computer simulations to uncover metabolic targets. A: Application of isoflurane reversibly accelerates FAD fluorescence decay as FAD-reduction. Flavin adenine dinucleotide (FAD) fluorescence averaged ± SD decay during 5minutes in control condition (black trace), under 3% isoflurane (red) and during wash out (green trace). Right on top: statistical comparison of the fluorescence percent decay in control (CTL), after treatment with 3% isoflurane and after wash out (single experimental values and medians). (b) Simulated changes of FAD reduction states for the pyruvate dehydrogenase- (PDHC), the α-ketoglutarate dehydrogenase- (KGDHC), the glycerol-3-phosphate dehydrogenase- (G3PDH_mito_), the succinate dehydrogenase (SUCCDH) complexes and ATP levels (left to right panels) for different degrees of inhibition of the respiratory chain complexes (CxI to CxIV, top row) and the citric acid cycle (CAC) enzymes PDHC, G3PDH_mito_, KGDHC, malate dehydrogenase (MDH) and citrate synthase (CS; bottom row). Inhibition of CxIII and CxIV of the respiratory chain leads to an oxidation shift in FAD in all enzymes, but only at enzyme inhibitions of more than 50%. CxI inhibition leads to an oxidation of FAD in PDHC and KGDH but a reductive shift of FAD in G3PDH_mito_ and SUCCDH even at low enzyme inhibition. Inhibition of CxII (SUCCDH) leads to increased FAD reduction in all enzymes. ATP availability is only compromised at very high enzyme inhibitions. Inhibition of CAC enzymes leads to a reduction shift in FAD in PDHC, KGDHC, G3PDH_mito_ and SUCCDH, with PDHC and CS inhibition having the most pronounced effect already at low enzyme inhibition. The black dotted line shows the reductive shift in FAD resulting from decreased ATP demand at 3% isoflurane administration. For experimental data, statistical comparison with paired t-test and Bonferroni correction, *p < 0.05, **=p < 0.01, n = 9.

**Figure 6. fig6-0271678X211010353:**
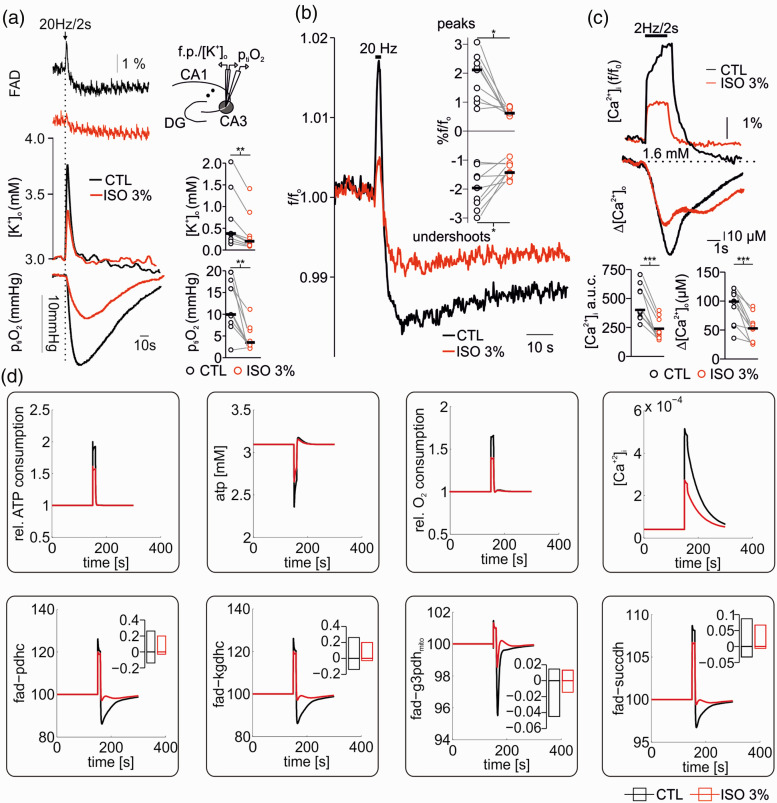
Effects of high concentrations of isoflurane on FAD changes associated to neuronal activation. (a) Simultaneous measurements of stimulus induced FAD, [K^+^]_o_ and p_ti_O_2_ changes in hippocampal slices before and after treatment with 3% isoflurane. Stimulus-induced [K^+^]_o_ rises and O_2_ consumption as well as the oxidative peak and the reductive undershoot of FAD decreased under 3% isoflurane. (b) Averaged stimulus induced FAD peaks and undershoots demonstrate a clear signal decay in the presence of 3% isoflurane. (c) Effects of 3% isoflurane on stimulus induced intracellular and extracellular calcium ([Ca^+2^]_i_ and [Ca^+2^]_o_) transients. Signals were simultaneously recorded by imaging with Oregon green (OGB-1) and using calcium sensitive microelectrodes. (d) Simulations of ATP consumption rate, ATP levels, relative O_2_ consumption rate, Ca^+2^ transients (upper panel) and corresponding FAD transients (lower panel) following electrical stimulus under CTL conditions (black) and with 3% isoflurane (red). Stimulation under CTL conditions was modelled by assuming a sudden increase in electrophysiological ATP demand by a factor of 3 together with an increase in cytosolic Ca^2+^ concentration to 4 µM due to membrane depolarization. At 3% isoflurane basal ATP demand was reduced to fit reduced O_2_ consumption rates and stimulus induced additional electrophysiological ATP demand was reduced by 38% as determined above (see Figure 1). Cytosolic Ca^2+^ transients were assumed to be reduced by 53% (see (c) and upper panel). The simulations show a reduced oxidation peek and a reduced oxidative shift in all FAD moieties at 3% isoflurane compared to control conditions. For experimental data, statistical comparison with paired t-test, *=p<0.05, **=p<0.01 and ***=p<0.005.

**Figure 7.**
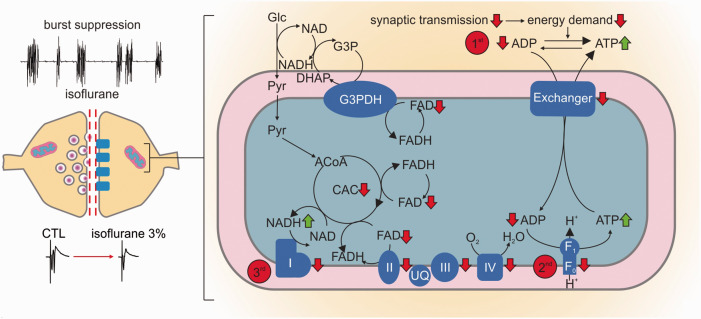
Changes in oxidative phosphorylation result from isoflurane induced decrease in synaptic activity. Left: During deep anaesthesia *in vivo* and *in vitro*, high dosed isoflurane induced a hypometabolic state characterized by the burst suppression pattern (*in vivo* data) and strongly decreased pre- and post-synaptic processes (*in vitro* data). Right (detail of mitochondrial processes): as synaptic processes are strongly inhibited, the following changes in the oxidative metabolism may occur: 1^st^: increase in ATP/ADP ratio with subsequent decrease in nucleotide exchanger activity; 2^nd^: as mitochondrial ATP concentration increases, the ATP-synthase is inhibited; 3^rd^: as less ATP is produced, the decrease in the proton gradient inhibits the respiratory chain complexes. Subsequently, mitochondrial NADH increases due to the inhibition of complex I. As a consequence, the pyruvate dehydrogenase (PDHC) and the α-ketoglutarate dehydrogenase (KGDHC) transfer electrons to NAD via enzyme bound FADH. Likewise, glycerol-3-phosphate dehydrogenase (G3PDH) and the complex II (succinate dehydrogenase, SUCCDH) transfer electrons to ubiquinone via enzyme bound FADH to the respiratory chain. The decrease in the respiratory chain activity leads to an increased reduction in ubiquinone (UQ), hampering the acceptance of electrons, which is again partially overcome by an increase in FADH bound to G3PDH and the complex II. For simplification the PDHC (between Pyruvate and Acetyl-CoA, abbreviation: ACoA) and KGDHC (in the citric acid cycle, abbreviation: CAC) are omitted in the diagram.

To distinguish cause and effect of reduced CMRO_2_ and neurotransmission, we studied changes in synaptic transmission and mitochondrial redox state by simultaneously recording neuronal activity and imaging FAD fluorescence baseline and stimulus induced FAD transients (Figures 5 and 6). FAD fluorescence intensity decreases continuously upon illumination of the brain slice due to photodecomposition and bleaching of the flavin chromophore.^24^ As the kinetics of bleaching is independent of the neuronal activity, acceleration or lowering the rate of fluorescence decay also reveals activity-dependent redox alterations in enzymes containing FAD as an electron donor.^25,30^ Application of isoflurane significantly accelerated the fluorescence decay from 1.7 (1.5,2.9)% under control condition to 4.3 (3.6,5.5)% for 3% isoflurane (decay measured in 5minutes, p = 0.03, n = 7, Figure 5(a)). As the reduced form FADH_2_ is less fluorescent, isoflurane generated a mitochondrial reductive shift, which was reversible upon wash out of isoflurane (after washout -1.8(-2.2,-1.0)%, p < 0.001, n = 7). We then performed computer simulations of the FAD baseline redox status for different hypothetical effects of isoflurane on respiratory chain complexes and key metabolic enzymes (Figure 5(b)). These simulations integrated the calculated changes in CMRO_2_ and ATP consumption rates assessed in prior experiments in slices (see Figure 2). Under the assumption that isoflurane has no direct effect on respiratory chain complexes, we predicted a reductive shift in the FAD redox state under 3% isoflurane as a consequence of decrease in ATP consumption (Figure 7). The resulting increase in the ATP/ADP ratio in the cytosol, reduces the activity of the adenine nucleotide translocator, which exchanges cytosolic ADP with mitochondrial ATP. Subsequently, the increased mitochondrial ATP concentration inhibits the activity of the ATP-synthase. As the ATP-synthesis utilizes the proton motive force generated by the respiratory chain, the decrease in ATP generation leads to an increase in the proton motive force (as it is now used to a lesser extend), which in turn inhibits the activity of the respiratory chain. Complex I of the respiratory chain uses electrons provided by mitochondrial NADH and a decrease in respiratory chain activity leads to an increase in mitochondrial NADH concentration. The PDHC and KGDHC transfer electrons to NAD via enzyme bound FADH. When the NADH concentration increases, this transfer is aggravated, but this aggravation is partially overcome by an increase in FADH reduction state. Likewise, G3PDH and SUCCDH transfer electrons to ubiquinone, an electron carrier of the respiratory chain, via enzyme bound FADH. Decreased respiratory chain activity leads to an increased reduction in ubichinone, hampering the acceptance of electrons, which is again partially overcome by an increased reduction of FADH bound to G3PDH and SUCCDH. Therefore and as observed in our FAD fluorescence measurements, the decreased ATP utilization induced by isoflurane administration leads to a reductive shift in FAD in all metabolic enzymes using FAD as a prosthetic group, i.e. in the PDHC, KGDHC, G3PDH and SUCCDH.

Next, we systematically decreased the activity of the respiratory chain complexes I to IV in our model and calculated the resulting FAD changes again adjusting the ATP consumption to the CMRO_2_ observed during isoflurane administration (Figure 5(b)). Increasing inhibition of complex I led to a strong oxidative shift in the FAD redox state of G3PDH and SUCCDH, while inhibition of complex II caused an oxidative shift in FAD redox state for PDHC, KGDHC, G3PDH, and SUCCDH. Inhibition of the complex III had little effects on redox state below 75% of inhibition and led to a reductive shift for PDHC, KGDHC, G3PDH and SUCCDH only at higher levels of inhibition which would decrease ATP availability impairing tissue viability which was not observed experimentally. Inhibition of complex IV had almost no effect on FAD reduction state. Taken in account that the observed FAD fluorescence is the result of changes in all moieties, a direct inhibition of mitochondrial complexes is not supported by our experimental data.

We further asked whether isoflurane could impair mitochondrial energy metabolism by inhibition of other key metabolic enzymes. Therefore, we systematically decreased the activity of the PDHC, citrate synthase (CS), KGDHC, malate dehydrogenase (MDH) and the G3PDH shuttle and simulated the FAD redox state. The inhibition of all tested enzymes except G3PDH led to oxidative shifts in the FAD redox state for PDHC, KGDHC, G3PDH and SUCCDH (Figure 5(b)) contradicting our empirical findings. As the malate aspartate shuttle can compensate for G3PDH inhibition, no effect in FAD redox state is computed.

These simulations showed that, as long the inhibition remains below 50%, a net reductive shift would occur, as observed in our experiments without impairment of neuronal energy metabolism (the ATP level would still be above 3 mM) (Figure 5(b), last panel). Stronger inhibition of respiratory chain enzymes would inevitably result in marked oxidative shifts (complex II) of FAD and severely reduce ATP levels contradicting the experimental findings (see Figure 6). Therefore, we conclude that even if there is a direct effect of 3% isoflurane on respiratory chain enzymes, it does not compromise energy metabolism. The same is true for inhibition of citric acid cycle enzymes since inhibition of PDHC, G3PDH, KGDHC, MDH or CS all result in an oxidative shift.

Lastly, we studied changes in FAD transients during sudden and strong increase in ATP demand due to stimulus-induced neuronal activation (Figure 6).^25^ The typical corresponding changes in FAD fluorescence after electrical stimulation (20 Hz/2 seconds in our protocol) are given by a short oxidative peak followed by a subsequent reductive undershoot (Figure 6(a) and (b)).^20,22,25^ The oxidative peak originates from the increased cytosolic ATP utilization, which increases the activity of the adenine nucleotide translocator and thereby decreases mitochondrial ATP concentration. This activates the ATP-synthase and the respiratory chain activity generating oxidation of mitochondrial NADH as well as ubichinone which increases FAD (oxidized form) bound to PDHC, KGDHC, G3PDH and SUCCDH, as electron transfer to NAD and ubichinone is eased. The electrical stimulus also increases the influx of Ca^+2^ into the mitochondria, which in turn activates the citric acid cycle by activation of key hydrogenases (PDHC, KGDHC and IDHC) leading to a recovery of FADH and NADH (reduced state). As the Ca^+2^ activation persists longer than the oxidative peak, there is a prolonged overshoot in NADH and FAD undershoot (FADH is not visible).

Experimentally, we monitored FAD transients, [K^+^]_o_ and p_ti_O_2_ during electrical neuronal activation upon 20 Hz train stimulation in control conditions and in the presence of 3% isoflurane. As shown in Figure 6(a) and (b), both components of FAD fluorescence transients decreased in the presence of isoflurane (the oxidation peak decreased from 2.1 (1.1,2.3) to 0.6 (0.5,0.8), p = 0.004, and the reductive undershoot decreased from − 1.9(1.5,2.4) to −1.4 (-1.5,-1.1), p = 0.01, for control and 3% isoflurane, respectively, n = 9). Simultaneously, stimulus-induced [K^+^]_o_ increase declined from 0.4 (0.25,0.7)mM in control condition to 0.2 (0.1,0.3)mM under 3% isoflurane (p = 0.007, n = 9) and p_ti_O_2_ dip during tetanic stimulation corroborated the decrease in O_2_ consumption under 3% isoflurane (Δp_ti_O_2_ CTL: 9.9 (8.0,15.0)mmHg and during 3% isoflurane: 3.4 (2.9,6.2)mmHg, p = 0.002, n = 9). In a complementary set of experiments, we investigated intra- and extracellular Ca^2+^ movements in the presence of 3% isoflurane. Here, we measured a significant decrease in stimulus induced [Ca^+2^]_i_ uptake (control Δf/f_o_ 389.9 (563.9,337.5) vs. 208.4 (318.9,163.1) under 3% isoflurane, p < 0.001, n = 10) and [Ca^2+^]_o_ decreases (99.0 (113.4, 58.3) µM in control vs. 52.8 (61.8,28.9) µM under 3% isoflurane, p < 0.001, n = 10, see Figure 6(c)).

Computer modelling of stimulus-induced FAD transients integrating calculated CMRO_2_, predicted changes in ATP and determined decreases in [Ca^+2^]_i_ as boundary conditions was performed as well (Figure 6(c)). The resulting simulations shown, in agreement with our experiments, that the decrease in FAD peak and undershoot component occurred without direct effect of isoflurane on enzymes of the respiratory chain or the citric acid cycle (Figure 6(b)).

## Discussion

In this study we investigated neuronal energy metabolism during deep isoflurane anaesthesia with an emphasis on potential direct effects of isoflurane on mitochondrial function. Our *in vivo* recordings indicated that the pattern of burst suppression is accompanied by dynamic changes in p_ti_O_2_ and rCBF. In brain slices, similar amounts of isoflurane to induce burst suppression strongly reduced synaptic transmission, network oscillations and Ca^2+^ influx into neurons, resulting in a significant CMRO_2_ decline. Assessment of FAD-autofluorescence revealed a reductive shift in mitochondrial redox state and decreased stimulus-induced FAD transients. Computational modelling based on our empirical data predicted no direct effects of isoflurane on mitochondrial enzymes.

### Cortical *p_ti_O_2_ and rCBF adapt to neuronal activity during deep anaesthesia with isoflurane*

During induction and establishment of burst suppression p_ti_O_2_ showed large variations between experiments and tended to slowly increase from physiological values of ∼28mmHg^27^ to ∼40mmHg while single-burst associated p_ti_O_2_ fluctuations occurred (Figure 1). Changes in cortical p_ti_O_2_ depend on fluctuations of rCBF limiting the possibility to assess the net O_2_ consumption by neurons (i.e. CMRO_2_). As shown in further experiments, burst suppression was associated with a decline in rCBF baseline (∼35%) combined with burst associated rCBF increases (Figure 2). In humans, decrease in CMRO_2_ occurred during deep isoflurane anaesthesia but results concerning total CBF were inconclusive. Whereas CBF calculations using arteriovenous O_2_ differences or Xenon-133 clearance suggested an increase of CBF,^28^,^29^ assessing CBF with Doppler blood velocity measurements combined with arteriovenous O_2_ differences during cardiopulmonary bypass rather suggested a decrease in CBF.^30^ Compared with other anaesthetics, isoflurane strongly reduced resting state functional connectivity (RSFC) using intrinsic optical imaging to assess blood volume.^31^ Using LDF with higher temporal and spatial resolution but limited to a small cortical region, we observed a general decrease in rCBF, paroxysmal increases in rCBF following bursts and, in line with previous reports,^32–35^robust stimulus-induced rCBF responses during deep anaesthesia. Thus, our findings suggest that during isoflurane induced burst suppression neuro-vascular coupling remained functional in the neocortex as microvasculature adapts to a lower energy demand (i.e. rCBF baseline) and short periods of energy use during bursts.^36–39^ Interestingly, rCBF decreased at the beginning of burst suppression, while p_ti_O_2_ baseline slowly increased ∼15minutes after the beginning of burst suppression. This seems to indicate that after an initial equilibrium between reduced O_2_ consumption by the tissue and reduced rCBF, O_2_ consumption continued to decrease with delay, while rCBF remained stable at a lower limit, which was not further undercut. However, caution is advisable with this interpretation, as measurements of p_ti_O_2_ and rCBF in the *in vivo* condition only allowed an indirect assessment of neuronal energy metabolism during isoflurane induced deep anaesthesia.

### Inhibition of synaptic transmission by isoflurane decreased energy demand in neurons

For measurements of isoflurane-induced changes in neuro-metabolism, we calculated CMRO_2_ under stable O_2_ and glucose supply while monitoring synaptic transmission and network oscillations *in vitro* (Figures 3 and 4). In naïve slices, isoflurane inhibited synaptic processes depending on the applied concentration which correlated with the decrease in CMRO_2_. The effects of 1% isoflurane in synaptic activation (decreased PS amplitude and increased PPR) suggested presynaptic mechanisms as described previously^17^,^40^ but generated little decrease in CMRO_2_. In the case of 3% isoflurane, PS and paired pulse facilitation were almost abolished indicating severe blockade of both, pre- and postsynaptic processes which correlated with significant decrease in CMRO_2_. During gamma oscillations-associated high metabolic demand, CMRO_2_ decreased in a concentration-dependent manner and, as in naïve slices, strongly correlated with synaptic inhibition, i.e. network activity impairment. Interestingly, we measured a higher CMRO_2_ after 3% isoflurane than prior to the induction of gamma oscillations. This suggests that blocking network activity by isoflurane was not related to limited oxidative phosphorylation in contrast to the situation during hypoxia or after application of the complex I inhibitor rotenone.^41,42^ Furthermore, isoflurane-dependent CMRO_2_ decrease was proportional to the degree of neuronal activity present in the slice . Isoflurane is known to inhibit pre- and postsynaptic targets, such as GABA_A_, NMDA receptors and presynaptic Ca^+2^ channels^40^ which account for significant portions of neuronal ATP consumption.^1^,^21^ Thus, our data suggest a decrease in ATP demand due to the inhibition of multiple processes involved in synaptic transmission.^21^ However, in the case of a direct alteration of mitochondrial functions by isoflurane, cellular respiration and ATP production would decrease thereby impairing chemical synapses. Indeed, direct blockade of the mitochondrial complex I impaired presynaptic transmitter release and has been proposed to play a central, receptor-independent role in isoflurane anaesthesia.^17,18^

### Discriminating between direct and indirect effects of isoflurane on mitochondrial function

We investigated possible effects isoflurane on ATP consumption and/or ATP synthesis by integrating experimental data and computational modelling. Assuming that ∼50% of the basal ATP consumption in neuronal tissue is accounted for electrophysiological processes,^21,25,43^ we predicted a decrease in ATP consumption by 16% and 38% at 1% and 3% isoflurane respectively (Figure 3). We further performed FAD imaging (changes in FAD baseline and in stimulus-induced FAD transients) to locate possible direct effects of isoflurane on the respiratory chain and mitochondrial key enzymes (Figures 5 [Fig fig6-0271678X211010353]to 7).^20,22,25,44^

Our computer simulations predicted that a decrease in ATP consumption alone would lead to the observed reductive shift in FAD fluorescence. As isoflurane has been shown to specifically inhibit mitochondrial complexes in neurons,^17^ we investigated whether a direct inhibition of mitochondrial complexes and key enzymes would be consistent with our FAD measurements. Our simulations revealed that moderate inhibition of respiratory chain enzymes would agree with the observed FAD reductive shift, while inhibition of the rate limiting enzymes CS and PDHC of the citric acid cycle can be excluded. Moreover, a severe inhibition of the ATP producing machinery would generate exactly the opposite, i.e. an oxidative shift in FAD. Thus, the good match between computational simulations, CMRO_2_ decrease and experimentally determined FAD suggested that highly concentrated isoflurane diminished ATP consumption. In line with our results, intracellular ATP increased during isoflurane anaesthesia in swine^19^ and the consequent indirect inhibition of the respiratory chain could explain a depolarisation of the mitochondrial membrane potential observed in previous studies.^45^

These assumptions were enforced by experiments concerning stimulus-induced FAD transients, [K^+^]_o_ increases, p_ti_O_2_ dips and subsequent Ca^2+^ movements (Figure 6). Here, [K^+^]_o_ increases (as a correlate of neuronal activation), p_ti_O_2_ dips, FAD transients and Ca^2+^ input onto neurons markedly decreased under 3% isoflurane indicating a decrease in oxidative phosphorylation. Simulations of sudden ATP use integrating known CMRO_2_ changes and measured Ca^+2^ movements reproduced the effects of isoflurane on FAD transients *in silico*. In conclusion, our data strongly suggests that isoflurane (at concentrations relevant for deep anaesthesia) blocked synaptic processes decreasing ATP use and generating secondary adaptations in mitochondrial function.

### Comparison to propofol, translational relevance and limitations

In previous studies it has been demonstrated that, in contrast to isoflurane, propofol directly alters the respiratory chain in neurons.^19,20^ Opposite to a reductive shift of FAD under isoflurane treatment, propofol induced an oxidative shift in our experimental *in vitro* conditions which was in line with an inhibition of the mitochondrial complex II when the same computational model was used.^20^ In the clinical setting, propofol is known to induce a systemic metabolic disorder (so called propofol infusion syndrome) and to unmask pre-existing mitochondriopathy.^46^ Surprisingly, propofol has become the most used anaesthetic for neurosurgical procedures and for sedation in neurocritical care, relegating gas anaesthetics to a third position after classical benzodiazepines and barbiturates.^13^

As severe brain diseases can be associated to profound alterations of neurovascular coupling up to inverse hemodynamic responses to the depolarization of principal neurons, anaesthetic induced changes in vascular reactivity and mitochondrial function might influence the chances of neurons to survive perfusion/metabolic mismatch.^7,37–39^ Additionally, long-term alterations in brain metabolism may contribute to the association of deep anaesthesia to postoperative complications.^47,48^ This evidences the need of a reliable assessment on neuro-metabolism before, during and after anaesthesia. Accordingly, our results concerning acute changes during burst suppression suggest that isoflurane might have a suitable metabolic profile in patients requiring deep anaesthesia. Thus, we need more experimental and clinical studies to understand the relevance, duration and precipitating factors of neurometabolic impairment (“chemical hypoxia”) potentially induced by anaesthetics.

Finally, our results demonstrate once again that anaesthesia represents a highly dynamic brain state with heterogeneous metabolic correlates depending on electrophysiological features. Thus, the performance of EEG-based neuromonitoring should be mandatory for patient-centred treatment during surgery and intensive care. The inclusion of appropriate methods for early detection of impaired neurovascular responses and mitochondrial dysfunction is likely to become increasingly important in the near future.

Of course, the effects of anaesthetics on neuronal function and cerebral energy metabolism may differ depending on species, age and studied brain region. In our study, we exploited the simple cytoarquitecture of the hippocampus to study changes in synaptic transmission, network activity and related changes in energy metabolism in vitro. As anaesthetics may differentially influence synaptic transmission and energy metabolism in the archicortex and the neocortex, further research are needed to identify possible regional differences in vascular reactivity, CMRO_2_ and mitochondrial function during anaesthesia.
